# The Physiology of Cataphoresis

**Published:** 1897-11

**Authors:** Weston A. Price

**Affiliations:** Cleveland, Ohio.


ARTICLE III.

### THE PHYSIOLOGY OF CATAPHORESIS.

BY WESTON A. PRICE, CLEVELAND, OHIO.

**Read before the American Dental Association at Old Point Comfort, Va., August, 1897.**

We will at this time confine ourselves to a consideration of the phenomena attending the application of an electric current to the human body, with and without an interposing medicament, and especially as applied to the dental organs.

There are three distinct theories as to the forces at work and their particular action in these processes, and and since there is such a diversity of nomenclature and variety of method for the application of these forces, we will include in this consideration all the methods of applying an electric current to the dental organs for producing anæsthesia, whether used in conjunction with a medicament or not. The theories are,—

1. The polarization of the tissue, producing an inhibition of the sensory impulses.
2. Osmosis.
3. Electrolysis.

The first theory provides that when an electric current is applied to a tooth with or without a medicament, the conditions produced are due entirely, or almost entirely, to the effects of the current, and not due to the medicament, other than its assistance in conducting a current. Another division of the first theory is that a constant current applied to the dental branches of the trifacial nerve, with the positive pole applied to the tooth and the negative pole over the Gasserian ganglion, inhibits the normal sensory impulse. The leading advocates of this theory maintain that a certain and definite amount of current applied in the manner just described will produce a condition of anæsthesia in the tooth, and that either too little or too much will not produce this condition. They call it "short-circuiting" the nerve. For this application, the positive pole of the constant current is attached to the dental engine in such a manner as to make the bur the electrode, the hand-piece being insulated, and the negative pole is applied over the Gasserian ganglion upon the same side as the tooth to be operated on, which is perfectly insulated.

The next theory provides that the medicament which is applied under the electrode is the agent which does the work, but that it is carried in by physical force, that in some way, just as a stream of water carries sediment with it, so the electric current carries the ingredients in solution with it through the solvent and through the tissue. The advocates of this theory have furnished almost all the literature that has been written on the theory of cataphoresis since its advent; and among its ranks are to be found many of the foremost men in the dental and medical professions. The most exhaustive articles bearing on this theory have been written by Drs. Morton, Peterson and Phillips, while a number of shorter articles have appeared from other writers.

The theory of electrolysis provides practically that all the effect produced in passing an electric current through a medicament, as applied in cataphoresis or in any other way is electrolytic. I have not been able to find a single person among the writers for the medical and dental profession defending this theory; it may, however, be worthy of consideration.

As the next steps let us consider,—

1. The physiological effect of a constant current on nerve tissue.

2. The laws governing osmosis.

3. The laws governing electrolysis.

I have demonstrated elsewhere, and verified repeatedly, that the resistance through a tooth, accordingly as the cavity is wet or dry, will vary all the way from thousandths to hundreds of thousandths of ohms. I have measured cavities in dentine after dehydrating, and found them to vary from twenty thousand ohms to over one million ohms, and in different parts of the same cavity almost that amount of variation over the surface of the dentine alone; through the enamel, of course, those figures would be multiplied by thousands.

Two things must be evident to every one at a glance, viz., that in delivering the current to a tooth from the bur as the positive electrode, it is impossible to have a uniform amount of current flowing, as the bur is moved to different parts of the cavity, owing to the variableness of the resistance of the different parts of the cavity, and that with so very high a resistance it would be impossible to have more than an extremely weak current flowing, unless the potential were very many times that used.

It has been demonstrated by Nernst and others that "the osmotic pressure is independent of the nature of the solvent, and, in general, obeys the laws of gases." Various proofs for establishing this law are given in Nernst's "Theoretical Chemistry," 1895. It has also been demonstrated that "solutions having the same osmotic pressure can be obtained by dissolving equimolecular quantities of the various substances in the same solvent."

Since the nature of the solvent has nothing to do with the osmotic pressure it at once becomes obvious that,—

1. It does not matter what solvent we use for our cocaine, providing it is the force of osmosis that accomplishes the work.

2. Since the osmotic pressure is in different proportion to the concentration, the solution should be as nearly saturated as possible.

3. Since the osmotic pressure is increased to a definite extent by each degree increase of heat, the solution should be kept as hot as possible.

These observations hold good for practical application, if the force we are dependent upon is osmosis. We can make our deductions both from a clinical and theoretical standpoint. Will osmosis carry cocaine into dentine to any considerable extent? To answer this, I have sealed a saturated solution of cocaine into cavities for two days, and again for two months, without producing anesthesia except on the very surface of the cavity. I have also applied it for some time on an exposed pulp, and could not go very far into it. Sulphate of strychnine and bichloride of mercury applied on cotton to the chest of frogs produced no physiological effect, while with a current, death was produced in a few minutes. This at one stroke seems to answer the question as to whether osmosis alone can produce this condition.

We are forced to conclude from the clinical observations and theoretical probabilities that osmotic pressure is not the force on which depends the transmission of cocaine through dentine into the pulp. This brings us to a consideration of the last question—viz, What are the laws of electrolysis?

What is electrolysis? It is the change that is effected by the passage of an electric current in so far as the electricity exhibits itself as such.

How is electrity conducted? As expressed by Nernst, and translated by Palmer, "The conveyance of electricity in conducting substances may happen in two different ways,—viz., with or without the associated transportation of matter. The latter happens in the case of metallic conductors (second class); hence these are called conductors of the first and second classes respectively."

A solution conducts electricity the better the more numerous the ions and the smaller the friction which the ions encounter in their migration. This conception may be applied unchanged to every substance which conducts electrolytically, whether gaseous, liquid, or solid, whether simple or mixed.

Let us now apply some of these laws of electrolysis to the particular process with which we are interested. Suppose the positive pole be applied to the dentine of a tooth and the negative to the cheek. An interposing layer of medicament, say cocaine, in watery solution, is between the metallic positive electrode and the dentine. The only way electricity can get from the metallic positive electrode to the metallic negative electrode is by the dissociation of some of the molecules, in every instance of the second class through which it passes. In every part of the course through the cocaine solution, the dentine, the pulp, the connective tissue, the blood-vessels, the muscular tissue, and the sponge on the negative electrode, there will be a cleavage of some of the molecules of the various chemical compositions into a positive and a negative ion. These ions with equal force and chemical equivalents start on their respective journeys towards their opposite poles. They meet with friction which varies from different ions, and since they have the same force behind them, pushing them, their velocities will vary with varied resistance. If in their course they meet a new ion, or an element or compound for which they have a greater affinity than the force which separated them, they will unite with it until they are again called into service. Unless an ion found such an affinity, it would keep on going until it got to the metal plate of the negative electrode, and if it could unite with it would do so; if not, it would be deposited upon it or be liberated in the form of gas.

According to the older theories, there was supposed o be a physical force exerted in some manner by the electric current, and this theory has yet its advocates. There is a newer theory, however, which is made by its introducer to explain all the phenomena. Its author is Nernst, of Gottingen, Germany. He maintains that there is no transmission of matter, except the ions themselves.

The final goal is, of course, to diminish the time. I do not believe this will be done by seeking directly for a substance that has a high osmotic pressure, but rather in seeking for a reaction that will produce the most active ion. It is true, however, that substances that have a high osmotic pressure have good conductivity Since the amount of current we can use is limited by the pain limit of the tissue, and the amount of electrolysis is a constant expression of the strength of current, of course the amount of chemical energy we can liberate is fixed, and we cannot hope to change this unless we can change some of the laws of their physiology, electrolysis, or chemistry.

We have left these unfixed conditions to modify,—viz., to select the ions with the greatest migration facility, and which themselves, or the compounds they will form, will produce greater physiological effect upon the tissue for the same unit of concentration in the tissue. There is no reason why great advancement should not be expected, and it is my opinion that when it does come it must come along this line.

I am indebted for valuable references to Professor Morley, of the Western Reserve University, Professor Miller, of Case School of Applied Science, and Professor Herdman, of the University of Michigan.—*International Dental Journal.*

